# High throughput prediction of chylomicron triglycerides in human plasma by nuclear magnetic resonance and chemometrics

**DOI:** 10.1186/1743-7075-7-43

**Published:** 2010-05-14

**Authors:** Francesco Savorani, Mette Kristensen, Flemming H Larsen, Arne Astrup, Søren B Engelsen

**Affiliations:** 1 Dept. of Food Science, Quality & Technology, Faculty of Life Sciences, University of Copenhagen, DK-1958 Frederiksberg C, Denmark; 2 Dept. of Human Nutrition, Faculty of Life Sciences, University of Copenhagen, DK-1958 Frederiksberg C, Denmark

## Abstract

**Background:**

The lipid content of the chylomicrons is a key biomarker and risk factor of cardiovascular diseases and for the understanding of obesity. A high throughput determination of chylomicrons in human blood plasma is outlined.

**Methods:**

The new method, which uses a combination of Nuclear Magnetic Resonance (NMR) analysis and multivariate calibration analysis (chemometrics), is based on a correlation analysis towards the established standard method (ultracentrifugation and colorimetric test kit) and enables extraordinarily fast, inexpensive, and robust prediction of triglyceride (TG) content in chylomicrons. It is the position and shape of the complex lipid methylene resonance band that determines the chylomicron TG status and this information is extracted by the multivariate regression method.

**Results:**

The resulting method is a relatively simple multivariate model that facilitates parsimonious and accurate prediction of chylomicron lipids from NMR spectra of blood. The chemometric model predicts the chylomicron TG content with a correlation coefficient (R) of 0.96 when plotted against density gradient ultracentrifugation data.

**Conclusions:**

The new rapid method facilitates large scale clinical and nutritional trials with inclusion of diagnostics of chylomicron status and thus creates new opportunities for research in lifestyle diseases and obesity.

## Background

Most of our current knowledge on the link between lipids, lipoprotein metabolism and cardiovascular disease (CVD) development relies on observations made on fasting individuals. Actually, we spend the vast majority of our time in a non-fasting postprandial state and recent studies indicate that plasma postprandial triglyceride (TG) concentrations are better at predicting risk of CVD than fasting levels [1]. When fat is absorbed in the human body, it enters the circulation in the form of intestinally derived TG-rich lipoproteins, essentially chylomicrons, usually within about 15 minutes after finishing a meal. The chylomicrons are released by exocytosis in the villi of the small intestine and are then secreted into the bloodstream at the thoracic duct's connection with the left subclavian vein. Chylomicrons are large micellar lipoproteins, having a diameter of 75 to 1200 nm, and are primarily composed of TG (85%) and contain some cholesterol and cholesteryl esters. They are surrounded by a surface monolayer of phospholipids, unesterified cholesterol and specific apoproteins. Until now Low Density Lipoprotein (LDL) cholesterol has been the primary suspect in the development of coronary artery disease [2]. However, it remains unclear why 40% of people who are highly vulnerable to suffering a stroke or heart attack have low or normal low density lipoprotein (LDL) levels. It is possible that chylomicron adhesion on arterial walls may be as important a risk factor as low density lipoprotein (LDL) cholesterol in causing strokes and heart attacks [3]. To determine an individual's ability to clear postprandial lipids or the capacity of a food ingredient to diminish lipid uptake, TG-rich lipoproteins and, in particular, chylomicrons can be measured after an oral fat load.

The "gold standard" analytical method for measuring TG concentration in chylomicrons and lipoproteins is fractionation by density gradient ultracentrifugation followed by colorimetric determination of TG (UC-TG) [4]. Unfortunately this is a time and labor-intensive method wherefore the number of samples that can simultaneously be analyzed is small and, as a consequence, the assessment of postprandial lipoprotein metabolism is rarely done in large clinical trials. However, as recently demonstrated [5,6], proton NMR spectroscopy and curve-fitting represent an alternative method for the measurement of postprandial TG-rich lipoproteins in humans.

Proton NMR (^1^H-NMR) spectroscopy has recently become an indispensable analytical technique for characterization of complex biological samples such as metabolite mapping in tissue and body fluids [7]. Lipoproteins have quite similar compositions and thus their subgroups give rise to very similar NMR signals. However, it has been shown that NMR signals of lipoproteins in blood are weakly shifted in frequency due to the different densities of the lipoproteins, giving rise to different local magnetic fields [8]. Extraction of quantitative information from NMR spectra is classically performed by curve fitting and/or integration of peaks [9-13]. However, in this study it is demonstrated that a multivariate pattern recognition approach, such as that employed for lipoprotein subgroups in human blood [14], can make quantization more efficient and provide robust and precise determinations. The mathematical basis for the fundamental advances in high throughput metabonomics [15] has largely been due to the use of multivariate models such as principal component analysis (PCA) [16] and partial least squares (PLS) regression [17], and facilitated by the ongoing computerized revolution.

In this study we demonstrate how NMR spectroscopy in combination with multivariate chemometric regression can be used to efficiently and accurately determine TG content of chylomicrons in human plasma. The results are regressed towards the ultracentrifugation reference method which represents the limiting step in exploiting the full potential of the new method here presented; for example, if the ultracentrifugation method is unable to perfectly separate the chylomicron fraction from the very close very low density lipoprotein (VLDL) one. To demonstrate the performance of the new method, human blood samples were obtained after ingestion of test meals varying in fiber content, as dietary fibers are known to affect postprandial lipemia [18].

## Methods

### Study design

The presented samples are a subset of human plasma samples which was part of a larger double-blinded randomized crossover single-meal study investigating the postprandial lipoprotein response as a function of dietary fiber intake, in which 18 young apparently healthy normal to moderately overweight males participated (age: 27.2 ± 4.0; BMI: 25.4 ± 2.2 kg/m^2^). No exclusion criteria were applied related to lipemia, but results indicate that subjects had normal to high cholesterol and TG plasma concentrations. Baseline blood samples (0 minutes) were drawn after an overnight fast. Hereafter, the subjects consumed one of four iso-caloric test breakfast meals (differing in dietary fiber content) within 20 minutes and additional blood samples were drawn at 30, 60, 120, 180, 240, 300 and 420 minutes. The blood samples were collected in tubes containing EthyleneDiamineTetraacetic Acid (EDTA) and placed on ice. The samples were centrifuged at 2800 rpm for 15 minutes at 4°C. Plasma was extracted and stored overnight at 4°C before NMR analysis. The large study design included a total of 576 plasma samples out of which 192 samples, equally representing each sampling time point, were selected for the UC-TG measurements for calibrating the PLS regression model. The clinical part of the study was carried out at the Department of Human Nutrition (University of Copenhagen) and was approved by the Municipal Ethical Committee of Copenhagen in accordance with the Helsinki declaration (KF 01-309595). The large study is described in more detail elsewhere (Kristensen *et al*., under review).

### NMR spectroscopy

Samples for NMR analysis were prepared by mixing 500 μl plasma and 60 μl D_2_O. ^1^H NMR spectra were acquired at 37°C on a Bruker Avance 500 spectrometer (Bruker Biospin GmbH, Rheinstetten, Germany) operating at a Larmor frequency of 500.13 MHz for ^1^H (11.75 Tesla) and using a ^1^H/^13^C Z-gradient 4 mm diameter, 120 μl active volume flow-probe. The spectra were recorded using the *zgcppr *Bruker standard pulse sequence. The relaxation delay was 5.0 s during which water presaturation was performed at 4.700 ppm. The 90 degree pulses had duration of 8.0 μs. The acquisition time for acquiring 32 k data point free induction decays was 1.573 s using a spectral width of 20.8278 ppm. A total of 64 scans were acquired and each free induction decay was zero-filled to 64 k points and apodized by a Lorentzian line-broadening of 0.3 Hz. Phase and baseline correction were performed manually. All spectra were referenced to the anomeric proton of -D-Glucose at 5.23 ppm. Processing was performed using Bruker Topspin™ 1.3 software. Due to occasional problems with the NMR flow probe, only 153 sample spectra out of the selected 192 were available for comparison with the UC-TG data.

### Ultracentrifugation

Chylomicron fractions were isolated by ultracentrifugation as described in details elsewhere [19]. In short, 3 ml of plasma for each sample was carefully overlaid with 2.5 ml salt solution of density 1.006 Kg/L in ultracentrifugation tubes which were then centrifuged for 23 minutes at 30000 rpm in an ultracentrifuge. The tubes were then sliced 45 mm from the bottom and the TG concentration was measured for the top-fraction, containing primarily chylomicrons and presumably minor amounts of very low density lipoproteins (VLDL), and for a corresponding plasma sample (total TG) by a colorimetric test kit (Roche TG, Roche Diagnostics GmbH, Mannheim, Germany). The intra- and inter-assay precisions were 0.6% and 1.4%, respectively. For reference purposes, total cholesterol was assessed using a colorimetric test kit (Roche TG, Roche Diagnostics GmbH, Mannheim, Germany), intra-assay precision is 0.9%, no inter-assay precision is provided as all samples for the same subject were analyzed in the same run. Analytical sensitivity for both TG and total cholesterol is 0.01 mmol/L.

### Multivariate data analysis

Multivariate data analysis (chemometrics) is a necessity to study the full dimension of NMR metabonomics data sets. Chemometrics is based on latent variables and facilitates an inductive exploratory strategy which is of fundamental scientific importance as a complement to the ubiquitous deductive research strategies [20]. For developing the new method, PLS regression [17] was used. This chemometric technique applies to the simultaneous analysis of two sets of variables on the same objects and is used in quantitative spectroscopy to correlate the spectroscopic data (X-block - fast multivariate measurements) to a univariate physico-chemical data (y-block - time consuming and laborious measurements). The main purpose of the regression analysis in this study is to build a model enabling the prediction of the reference UC-TG characteristic (y) from a measured NMR spectrum (x). In matrix notation the linear model y = Xb is obtained, where b contains the regression coefficients that are determined during the calibration step. The PLS-calculation starts with a covariance based calculation and, when using mean centered data, the elements of the first latent PLS-factor (calculated as X'y) will be proportional to the covariance of the spectral intensities of X with respect to the y vector of reference measurements. This illustrates that the information in the X matrix will be extracted guided, or supervised, by the information in y. PLS can be applied to the entire NMR spectra as it has been previously done to lipoprotein subgroups in human plasma [14] but, in order to develop a more parsimonious regression model, the interval partial least squares method (iPLS) [21] was applied. This approach has previously proven to be successful for the determination of cholesterol in rodent plasma lipoprotein fractions [22]. The method is identical to PLS but divides the spectrum into a number of subintervals for each of which is developed a unique PLS regression model. In this study all reported PLS models are validated using full leave-one-sample-out-at-a-time cross validation in order to avoid overfitting and to present robust results. All PLS and iPLS models were performed in Matlab^® ^(2007b, The Mathworks Inc., Natick, MA, USA) using the *i*toolbox available at http://www.models.life.ku.dk/source/.

## Results

### NMR peak assignment

**Figure 1:**
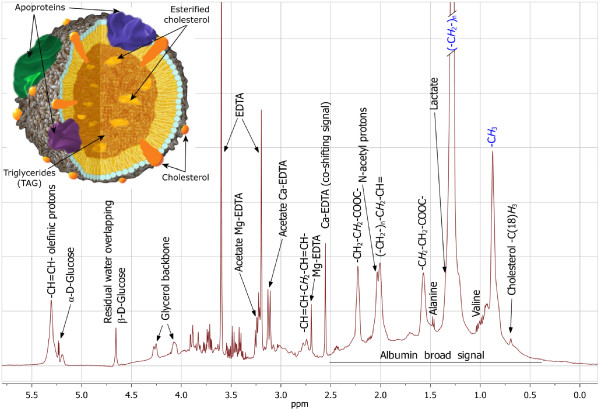
**Plasma ^1^H NMR spectrum**. A representative 500 MHz ^1^H NMR spectrum of human blood covering the entire lipid region from 0.0 ppm to 5.5 ppm. Inset shows a schematic view of the micellar chylomicron structure. The principal structures are indicated.

A representative NMR spectrum (0.0-5.8 ppm) of human plasma is shown in Figure 1, including peak assignment of all the significant resonances. This region of the spectrum shows all the broad peaks of lipoprotein TG, from the strong methyl and methylene resonances centered at 0.87 ppm and 1.28 ppm, respectively, to the broad olefinic resonance at 5.30 ppm, with the characteristic overlap of the alpha-anomeric proton doublet signal from the blood glucose. Cholesterol methyl resonance is visible at the right sidelobe of the methyl peak at 0.69 ppm. Besides the lipid signals, the considered region contains information from the blood sugars, the peptide side-chains and from EDTA, used to prevent plasma from coagulating.

### Partial Least Squares calibration

Table 1 sums up the ultracentrifugation results and shows that the chylomicron TG content of the samples used in this study ranges from 0.08 to 2.76 mmol/L providing a reasonable range for calibration of the PLS regression model.

**Table 1:** Sample overview.

	Average (mmol/L)	Standard dev. (mmol/L)	Min (mmol/L)	Max (mmol/L)
Fasted chylomicron TG (*n = 20*)	0.41	0.22	0.10	0.92

Postprandial chylomicron TG (*n = 133*)	0.81	0.57	0.08	2.76

Plasma chylomicron TG (*n = 153*)	**0.76**	**0.55**	**0.08**	**2.76**

Plasma total cholesterol	4.43	0.80	3.30	6.43

Chylomicron TG content for the 153 plasma samples used for the PLS regression including all sampling time points. Ultracentrifugation results are shown for both fasted, postprandial and all samples (fasted + postprandial). The average, the standard deviation and the range of each subgroup are presented. Additionally, also the amount of total cholesterol is provided for the same samples. Accuracy of measurements is 0.01 mmol/L for the colorimetric methods.

The PLS calibration model resulting from the selected 153 samples using the entire NMR spectral information to develop the PLS model yielded a resulting correlation coefficient towards the UC-TG concentration, R^2^, of 0.90 and a root-mean-square-error-of-cross-validation (RMSECV) of 0.174 mmol/L TG using just 3 principal components. While this result is exceptionally encouraging, an attempt to improve the model and its interpretation was made by application of iPLS regression. In this extension to PLS the NMR spectra are divided into a number of small regions or intervals for each of which a local PLS regression model is calculated.

**Figure 2:**
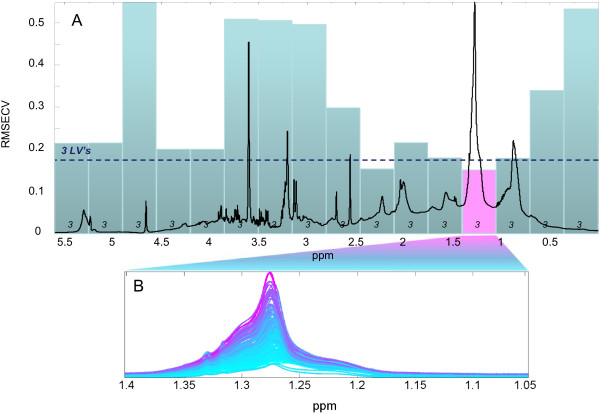
**iPLS statistical model**. Overview of the interval PLS regression model (A). The complex distributed methylene resonance is selected and provides a performance superior to the overall calibration obtained using the full NMR spectrum. Inset B shows the methylene peak colored according to the sample content of chylomicron TG. Higher concentrations are slightly shifted towards higher chemical shift values (ppm).

**Figure 3:**
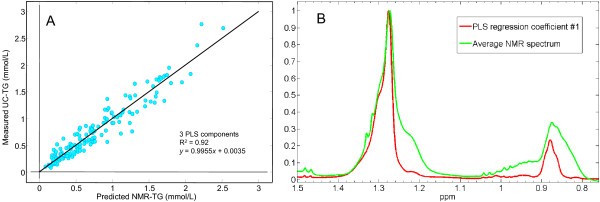
**PLS regression performance**. Cross-validated PLS regression model performance as viewed by a predicted (NMR-TG) versus measured (UC-TG) plot for the 153 plasma samples (A). This 3-component model is based on the spectral region 1.05-1.4 ppm and gives a correlation, R^2^, of 0.92 and a RMSECV of 0.156 mmol/L. The equation of the obtained linear regression is y = 0.9955x + 0.0035 mmol/L. The main lipid spectral region and its PLS regression coefficients are plotted superimposed (B) showing that only the left part of both the methylene and methyl peaks are informative for Chylomicron TG determination.

Figure 2A outlines the iPLS regression model to chylomicron TG: the horizontal dashed line shows the performance of the full-spectrum regression model (R^2 ^= 0.90 and RMSECV = 0.174 m mol/L TG using just 3 PLS components as mentioned above) and the interval-bars show the RMSECV performances of the spectral-interval regression models. Evidently, two spectral regions can further optimize the regression model: one region which includes the vicinal methylene groups to the ester groups of the TG and one region which includes the (poly)-methylene protons centered at 1.28 ppm. Figure 2B shows the 153 superimposed NMR spectra in the methylene region colored according to the sample UC-TG values. The figure reveals that the methylene peak shifts towards higher ppm with higher UC-TG content indicating that no simple univariate model can provide a comparable prediction model. The local PLS model performance for the methylene peak, which gives the best and most parsimonious model for predicting the chylomicron TG content, is shown in Figure 3. Figure 3A shows the PLS predicted and validated values of TG content plotted against the ultracentrifugation measured ones. The point-scatter around the ideal target line (x = y) demonstrates high linearity throughout the TG range and an unusual high NMR correlation to chylomicron TG. Indeed, the obtained regression model has an R^2 ^of 0.92 and a prediction error RMSECV of 0.156 mmol/L TG using only 3 PLS components. This result is to be compared to the standard deviation of 0.55 mmol/L TG obtained on the UC-TG reference method and indicate that the iPLS model provides a near perfect correlation to the UC-TG data. Additional information can be derived by inspection of the PLS regression coefficients. Figure 3B shows a superimposition of an average plasma NMR spectrum and the corresponding PLS (#1) regression coefficients for the main lipid spectral region (1.5-0.8 ppm). This comparison reveals that it is mainly the left part of both the methylene and the methyl peaks which is relevant for chylomicron TG determination, in agreement with observations made from Figure 2B.

### Time course study

**Figure 4:**
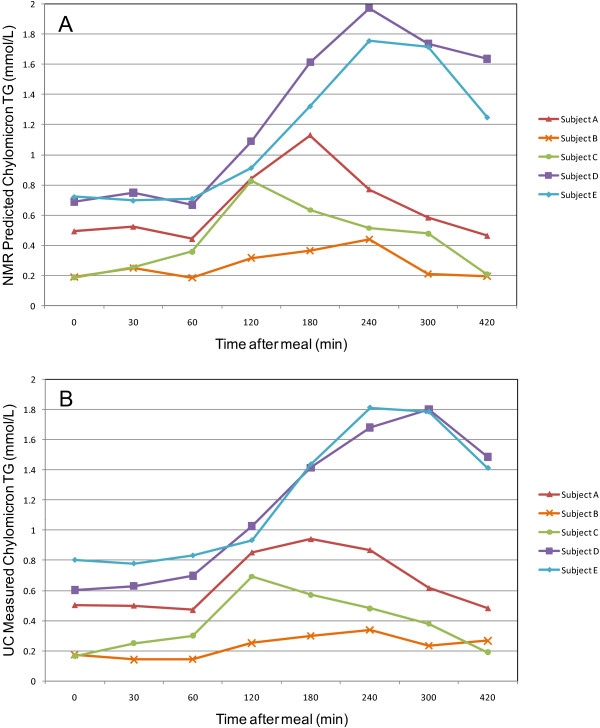
**Time course study**. Chylomicron TG concentration level for 5 subjects as a function of time after intake of a standardized meal. (A) The PLS predicted values are plotted against 8 time points spanning 7 hours. (B) The reference TG results by the ultracentrifugation method. The highest concentration is reached between 120 and 240 min after meal.

In order to demonstrate how the presented method can be used to monitor changes in plasma chylomicron TG concentration as a function of time elapsed since intake of a standardized meal, a simple time course study was carried out for five individuals. In Figure 4A the results obtained by the new method are shown for 8 time points from 0 to 7 seven hours after intake of the meal. For reference, also the UC-TG results for the same time courses are shown in Figure 4B. According to the analysis shown above, the differences between the ultracentrifugation and the NMR methods are at least partially due to the much higher uncertainty of the ultracentrifugation method. Nevertheless, the figure demonstrates the validity of the new method in monitoring postprandial TG concentration time changes occurring for different subjects.

## Discussion

The new method for determination of chylomicron TG concentration offers a low sample volume, high throughput and less expensive method that can allow extensive measurements in large clinical metabonomics trials and in future individual diagnostics. The time that elapses from when the blood is sampled to when the result is produced is reduced from several hours (ultracentrifugation) to minutes with the new method (NMR). The results achieved are consistent with previous NMR based methods [5,6,8] even though the new method avoids the use of curve fitting and peak integration steps, keeping the analysis simple and robust.

The iPLS approach, which for the first time is applied to the determination of chylomicron TG, results in a simple model which focuses on the most informative region of the spectrum and will thus be less sensitive to variations occurring on the overall metabolic profile.

The presented method can, with some additional calibration efforts, be used for robust and automatic prediction of the chylomicron TG plasma content as a routine analysis in hospitals and as a research tool for studying large scale clinical investigations and detailed postprandial time trials.

When considering potential interferences for the new NMR-based method, it should be emphasized that the method is sensitive to methylene groups and their environment. This mean all long chain fatty acids present in the chylomicrons, independently if they are part of phospholipids or diglycerides etc. Nevertheless, the spectral features of cholesteryl esters and other rare fats in the chylomicrons, that potentially can contribute to the methylene resonances, are quantitatively outnumbered in the supervised analysis and the high correlation to UC-TG is the proof. The method's sensitivity to TGs in chylomicrons is due to the slow diffusion of the large chylomicron particles which affects the shape and position of the methylene resonance. This NMR information can be used to calculate the diameter of the chylomicron particles as done by Dyrby et al. [23], but in this supervised study this information is implicitly used.

Since UC-TG is used as response in the regression analysis, and since this method does not allow a perfect separation between chylomicrons and very low density lipoproteins (VLDL), the new method, which performs correlation analysis, will per definition also be affected by the presence of this last class of lipoproteins when assessing the chylomicron TG content. If the inclusion of minor fractions of very low density lipoproteins (VLDL) in the correlation analysis is a problem, it should result in fasted plasma samples with a significant content of chylomicron TG. However, this is clearly not the case as the fasted plasma samples are all well predicted by the new method and do not compromise the linearity of the relationship between NMR spectral features and TG content. Albeit the concentration of chylomicron TG in the fasted state should theoretically be 0 mmol/L, it is well known that some individuals do not clear lipids at the same high rate, and that chylomicrons may be present even after an overnight fast [19]. Furthermore, a few subjects had both high fasting total TG (~2 mmol/L) (data not shown) and chylomicron TG (~0.9 mmol/L) indicating poor lipid clearance capacity, resulting in a relatively high mean fasting chylomicron TG concentration.

Finally, the time course study conducted on five subjects demonstrated good potential of the new method for monitoring variations in chylomicron TG concentration over time which is comparable or better to what can be obtained using the ultracentrifugation method. Most importantly, the low sample volume requirements and high throughput capacity of the new method will allow much more frequent time sampling for studying early events and finer details in the time courses for postprandial triglyceride (TG) concentrations after intake of standardized meals.

Due to the escalating obesity epidemic and its cardio-metabolic complications, in combination with the established relationship between postprandial elevation of lipids and CVD risk, the search for food ingredients and drugs that effectively suppress postprandial lipemia has increased in recent years. To determine an individual's ability to clear postprandial lipids or the capacity of a food ingredient to diminish lipid uptake, TG-rich lipoproteins and, in particular, chylomicrons must be measured after an oral fat load.

## Conclusions

The new method facilitates rapid measurement of chylomicron TG and creates new opportunities for research in lifestyle diseases and obesity and may become a valuable tool in nutritional research for assessment of absorption of exogenous diet-derived lipids. Furthermore, non-fasting concentration of TG in TG-rich lipoproteins including chylomicrons may replace fasting levels in assessing CVD risk once standard reference values have been developed, and thus a high throughput NMR method may become a valuable tool in prediction of disease risk.

## List of abbreviations used

CVD: Cardiovascular disease; UC-TG: Ultracentrifugation-Triglycerides; NMR: Nuclear Magnetic Resonance; PLS: Partial Least Squares regression; iPLS: interval-PLS; RMSECV: Root Mean Square Error of Cross Validation.

## Competing interests

The authors declare that they have no competing interests.

## Authors' contributions

FS and SBE performed the NMR data analysis, the multivariate statistical analysis and data interpretations. FHL performed the NMR measurements. AA designed the human intervention study and MK performed the reference TG chylomicron measurements. FS and SBE made the figures. FS, SBE and MK wrote the manuscript. All authors have read and approved the final manuscript.
